# “It Is a Tiger That Devours Me, But I Am the Tiger”

**DOI:** 10.3201/eid2712.AC2712

**Published:** 2021-12

**Authors:** Byron Breedlove

**Affiliations:** Centers for Disease Control and Prevention, Atlanta, Georgia, USA

**Keywords:** art science connection, emerging infectious diseases, art and medicine, about the cover, public health, severe acute respiratory syndrome coronavirus 2, SARS-CoV-2, coronavirus, viruses, coronavirus disease, COVID-19, respiratory infections, bacteria, It Is a Tiger That Devours Me, But I Am the Tiger, The Tiger, Franz Marc, animals, tigers, bacteria, One Health, infectious diseases, wildlife, zoonoses

**Figure:**
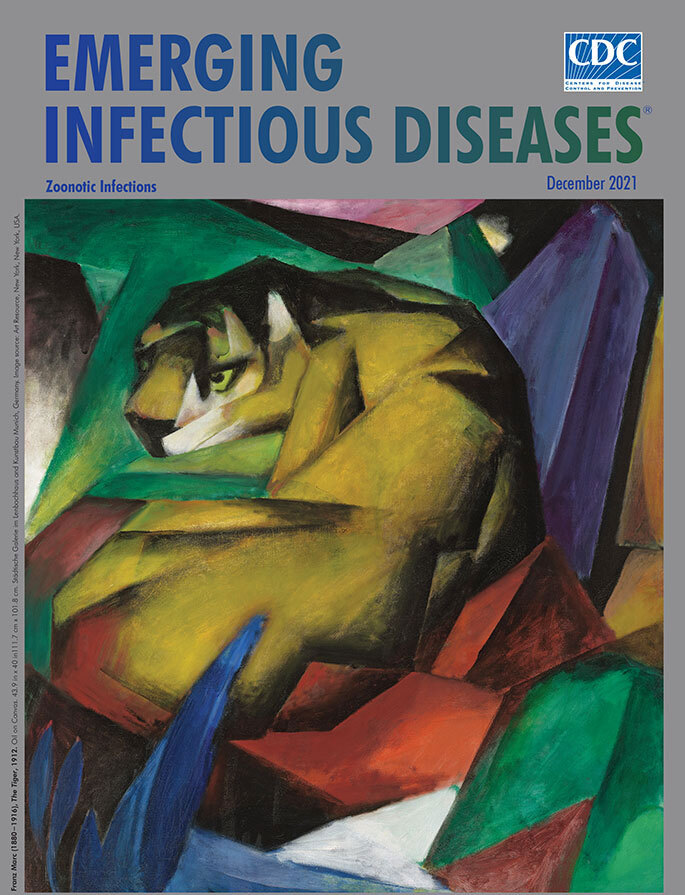
**Franz Marc (1880−1916), The Tiger, 1912.** Oil on canvas. 43.9 in x 40.0 in/111.7 cm x 101.8 cm. Städtische Galerie im Lenbachhaus and Kunstbau, Munich, Germany. Image source: Art Resource, New York, New York, USA.

On March 11, 2020, the World Health Organization declared COVID-19 to be a pandemic. Shortly thereafter, reports of animals becoming infected with SARS-CoV-2 appeared. Although the pandemic is being driven by person-to-person transmission, transmission from people to animals of multiple species has been documented. According to the Centers for Disease Control and Prevention, the animals known to have been infected with SARS-CoV-2 include otters, mink, white-tailed deer, dogs, ferrets, and felids, including domestic cats, lions, pumas, and tigers. 

An April 2020 US Department of Agriculture statement concerning a New York zoo’s lions and tigers showing clinical signs of respiratory illness was among the earliest such reports. Diagnostic samples taken from one tiger confirmed infection with SARS-CoV-2, and public health officials postulated that the source was exposure to a zoo employee positive for the virus. Stories about captive great cats with clinical signs of respiratory illness testing positive for SARS-CoV-2 have continued generating headlines from diverse locations around the globe. 

Reports of animals becoming infected with SARS-CoV-2 through contact with humans may be in the spotlight, but humans are also disease vectors for numerous other pathogens. A 2014 literature review in *PLoS One* documents myriad cases in which humans transmitted influenza A virus, *Mycobacterium tuberculosis*, methicillin-resistant *Staphylococcus aureus,* and other pathogens to animals and stated that “transmission occurred in every continent except Antarctica therefore indicating a worldwide disease threat.” A report by Iatta et al. in the *International Journal for Parasitology: Parasites and Wildlife* states, “Infectious diseases by pathogens, including those of zoonotic concern, may act as a primary or contributory cause of threat to wildlife conservation and may represent a risk for human health, mainly for people working at, or visiting the zoological parks.”

That message is underscored by accounts of captive great cats becoming infected with SARS-CoV-2. More tigers now live in captivity than in their natural habitats, putting them at potential risk of acquiring infections from people. Actions that are based on One Health and that recognize that the health of humans, animals, and the environment is closely connected will be increasingly important for ensuring the survival of animals of keystone species, such as tigers, as well as in helping to disrupt the cycle of transmission for zoonotic pathogens, and in increasing understanding of One Health issues across disciplines and sectors. 

This month’s cover image, *The Tiger*, is by German Expressionist artist Franz Marc. He was the son of a landscape painter, and he studied at the Academy of Fine Arts, Munich. In 1903 and 1907, he traveled to Paris, where he learned about Japanese woodcuts and the art of the Impressionists, Cubists, and Expressionists. Marc, along with Russian artist Wassily Kandinsky, founded the avant-garde group *Der Blaue Reiter* (The Blue Rider). Many of Marc’s works completed during his short life―he was killed during combat in World War I―vividly depict animals. The Brooklyn Museum notes that he “cultivated a dynamic Expressionist style that used rhythmic patterns of color and line to evoke movement.” In Marc’s own words, he wanted to “achieve a pantheistic empathy with the throbbing and racing of the blood in nature, in trees, in animals, in the air.”

The Lenbachhaus Museum, which houses the painting, notes that the “almost square image format is dominated by the mighty, crouching form of a tiger, which, with angular outlines as if carved out of stone, turns its beautifully shaped head back in a bold swing.” Marc used interlocking, bold blocks to form the tiger’s yellow and black body. The tiger’s diamond shaped eyes transfix the viewer. The landscape surrounding the tiger comprises angular, cubic forms that Marc imbues with rich, glowing shades of red, green, violet, and orange. The image bristles with tension and energy, as though its fractured components could suddenly coalesce and lunge snarling from the canvas. 

The Lenbachhaus offers additional insight into Marc’s tiger: “The facets of his glowing yellow body join with the transparent, cubic formations of his surroundings to form an indissoluble unit in which there is no longer any distinction between organic and inorganic substances.” The viewer, captivated, gains some knowledge of the tiger’s perspective, perhaps feeling both discomfort and connection. Argentine writer Jorge Luis Borges concludes his mid-1940s essay, “A New Refutation of Time,” with the following lines that both evoke the One Health perspective on the interconnectedness of humans, animals, and nature and that, in recalling Marc’s notion of pantheistic empathy, could serve as a caption for Marc’s The Tiger: “Time is the substance from which I am made. Time is a river which carries me along, but I am the river; it is a tiger that devours me, but I am the tiger; it is a fire that consumes me, but I am the fire.”
